# Identification of Psycho-Socio-Judicial Trajectories and Factors Associated With Posttraumatic Stress Disorder in People Over 15 Years of Age Who Recently Reported Sexual Assault to a Forensic Medical Center: Protocol for a Multicentric Prospective Study Using Mixed Methods and Artificial Intelligence

**DOI:** 10.2196/46652

**Published:** 2023-10-16

**Authors:** Emma Fedele, Victor Trousset, Thibault Schalk, Juliette Oliero, Thomas Fovet, Thomas Lefevre

**Affiliations:** 1 Institute for Interdisciplinary Research on Social Issues (UMR 8156) Aubervilliers France; 2 Department of Health, Medicine and Human Biology Sorbonne Paris Nord University (Paris 13) Bobigny France; 3 Department of Legal and Social Medicine Jean Verdier Hospital Assistance Publique - Hôpitaux de Paris (AP-HP) Bondy France; 4 Lille Neuroscience & Cognition Research Center Regional University Hospital of Lille University of Lille Lille France

**Keywords:** sexual violence, posttraumatic stress disorder, functional outcomes, risk factors, artificial intelligence, trajectory, longitudinal, mixed methods, sexual assault, mental health, cohort study, PTSD, innovative

## Abstract

**Background:**

Sexual assault (SA) can lead to a range of adverse effects on physical, sexual, and mental health, as well as on one’s social life, financial stability, and overall quality of life. However, not all people who experience SA will develop negative functional outcomes. Various risk and protective factors can influence psycho-socio-judicial trajectories. However, how these factors influence trauma adaptation and the onset of early posttraumatic stress disorder (PTSD) is not always clear.

**Objective:**

Guided by an ecological framework, this project has 3 primary objectives: (1) to describe the 1-year psycho-socio-judicial trajectories of individuals recently exposed to SA who sought consultation with a forensic practitioner; (2) to identify predictive factors for the development of PTSD during the initial forensic examination using artificial intelligence; and (3) to explore the perceptions, needs, and experiences of individuals who have been sexually assaulted.

**Methods:**

This longitudinal multicentric cohort study uses a mixed methods approach. Quantitative cohort data are collected through an initial questionnaire completed by the physician during the first forensic examination and through follow-up telephone questionnaires at 6 weeks, 3 months, 6 months, and 1 year after the SA. The questionnaires measure factors associated with PTSD, mental, physical, social, and overall functional outcomes, as well as psycho-socio-judicial trajectories. Cohort participants are recruited through their forensic examination at 1 of the 5 participating centers based in France. Eligible participants are aged 15 or older, have experienced SA in the last 30 days, are fluent in French, and can be reached by phone. Qualitative data are gathered through semistructured interviews with cohort participants, individuals who have experienced SA but are not part of the cohort, and professionals involved in their psycho-socio-judicial care.

**Results:**

Bivariate and multivariate analyses will be conducted to examine the associations between each variable and mental, physical, social, and judicial outcomes. Predictive analyses will be performed using multiple prediction algorithms to forecast PTSD. Qualitative data will be integrated with quantitative data to identify psycho-socio-judicial trajectories and enhance the prediction of PTSD. Additionally, data on the perceptions and needs of individuals who have experienced SA will be analyzed independently to gain a deeper understanding of their experiences and requirements.

**Conclusions:**

This project will collect extensive qualitative and quantitative data that have never been gathered over such an extended period, leading to unprecedented insights into the psycho-socio-judicial trajectories of individuals who have recently experienced SA. It represents the initial phase of developing a functional artificial intelligence tool that forensic practitioners can use to better guide individuals who have recently experienced SA, with the aim of preventing the onset of PTSD. Furthermore, it will contribute to addressing the existing gap in the literature regarding the accessibility and effectiveness of support services for individuals who have experienced SA in Europe. This comprehensive approach, encompassing the entire psycho-socio-judicial continuum and taking into account the viewpoints of SA survivors, will enable the generation of innovative recommendations for enhancing their care across all stages, starting from the initial forensic examination.

**International Registered Report Identifier (IRRID):**

DERR1-10.2196/46652

## Introduction

### Background

In recent years, sexual assault (SA), defined by French law as any form of sexual abuse involving violence, coercion, threats, or surprise, and occurring without clear and explicit consent, has gained recognition as a public health issue. Research indicates high prevalence rates of SA [1] and highlights its association with numerous negative outcomes, including effects on physical, sexual, and mental health; social life; financial stability; and overall quality of life [2]. It is recognized as one of the most traumatic events [3-5]. However, not everyone who experiences SA will necessarily develop negative functional outcomes. Various protective and risk factors can influence their psycho-socio-judicial trajectories. One of the most comprehensive models for categorizing these factors is the ecological model developed by Campbell, Dworkin, and Cabral [6]. This model is based on Ecological Theory [7], which classifies factors according to the environment in which they develop in relation to the individual.

Although there is a growing body of literature on the characteristics associated with negative outcomes and the utilization of medical, social, and legal resources, the precise manner in which these characteristics influence trauma adaptation remains unclear and does not enable the prediction of psycho-socio-judicial trajectories. Following an ecological framework, this project aims to advance research in this area using mixed longitudinal data and artificial intelligence (AI) to delineate the 1-year psycho-socio-judicial trajectories of individuals who recently experienced SA. Additionally, it seeks to identify predictive factors for the development of posttraumatic stress disorder (PTSD). Furthermore, it serves as an exploratory study of the needs and experiences of people who experienced SA and the available resources for them in France.

### Sexual Assaults and Negative Functional Outcomes

Data from the Global Burden of Disease study revealed that the global prevalence of SA in 2017 was 11.6% for men and 27.5% for women [1]. However, prevalence rates vary greatly depending on the definition of SA adopted by researchers, the research settings, and the study populations. A review of literature on SA prevalence outside of North America since 2010 revealed that past-year prevalence rates ranged from 0% to 59.2% for women, 0.3% to 55.5% for men, and 1.5% to 18.2% for individuals identifying as lesbian, gay, bisexual, and gender-diverse [8].

The literature demonstrates that SA can have a substantial impact on the quality of life and functional outcomes, affecting physical and sexual health, behaviors, financial stability, social life, as well as psychological and psychiatric well-being [2,9-11]. Among the various outcomes of SA, psychological and psychiatric effects have been extensively studied, with a particular focus on PTSD due to its strong association with SA. Indeed, US and international population–based studies indicate that the lifetime prevalence of PTSD ranges from 4% to 7% among individuals who have experienced any type of traumatic event [3-5]. However, for those who have endured rape and other forms of SA, these lifetime prevalence rates vary significantly, ranging from 17.4% to 65% [3-5], highlighting SA as one of the most profoundly traumatic experiences.

### Factors Associated With SA Negative Outcomes

Functional outcomes can vary considerably and are influenced by a multitude of protective and risk factors. Campbell, Dworkin, and Cabral [6] developed a framework to examine the diversity of these factors [6]. This framework is based on Bronfenbrenner’s Ecological Theory [7], which states that human development “evolves as a function of the interplay between person and environment” [7]. The authors adapted this framework to comprehend “how factors at multiple levels of the social ecology contribute to (...) deleterious mental health effects” [6]. It is one of the most comprehensive models to date as it classifies protection and risk factors into 7 levels depending on the environment in which they interact with the person. Using the same levels as defined by Campbell, Dworkin, and Cabral [6], we included in this review factors associated with negative mental health outcomes, overall recovery, and quality of life.

Individual-level factors represent the intrinsic characteristics of individuals that can affect their mental health and recovery after SA [6]. These are sociodemographic factors [6,11-16], preexisting mental health conditions and family history of mental health problems [6,17-19], and coping strategies [6,20-23]. Coping strategies encompass both positive strategies such as religious coping [24,25] and maladaptive strategies such as self-blame, which is related to more PTSD and depressive symptoms [6,20,26]. Campbell, Dworkin, and Cabral [6] conceptualized this last factor as a meta-construct, arguing that it transcends any single level of the model as it is influenced and enhanced by factors across all other levels.

Characteristics of the assault, such as the perceived life threat during the assault and the perceived dangerousness of the assailant, have been linked to negative mental health outcomes, as evidenced by research on SA [6]. These factors are also associated with general trauma [18,19]. Peritraumatic factors, including peritraumatic distress and dissociation, may also serve as moderate to strong predictors of PTSD, especially in cases of interpersonal violence [18,19], although documentation of these factors is relatively scarce.

The microsystem level is defined as the “face-to-face interactions and interrelations between individuals and others in their immediate setting” [6,7]. It encompasses factors such as a lack of social support, which is considered one of the most important risk factors for mental disorders [18,19,27]; and negative reactions following disclosure, which can severely impede recovery [6,27] and are associated with posttraumatic distress [28] and self-blame [29]. By contrast, positive social reactions are shown to lower psychological distress [6] and are associated with positive life changes [28].

The meso/exosystem level, as defined, involves interactions with the formal system [6]. This encompasses both positive formal support and instances of secondary victimization within the legal, medical, and mental health systems, as well as within the advocacy community. Perceptions of social reactions from formal responders have been associated with psychopathology [6,27]. However, results are not always consistent between studies: the only study to our knowledge to compare formal and informal support showed that reactions from formal providers, regardless of whether the reaction was positive or negative, were not associated with more general psychological distress; however, positive reactions were associated with benefits in the aftermath of trauma [28]. Regarding various forms of formal support, the literature indicates that the legal system tends to have the most adverse impact on mental health due to the prevalence of questions blaming the impacted individual. Additionally, the medical system has been shown to exacerbate PTSD symptoms [6,30]. By contrast, the mental health system and the advocacy community have been found to be supportive and beneficial for individuals who have experienced SA, especially those who have had negative experiences with the legal and medical systems. These entities can help mitigate the adverse effects of such experiences [6].

The macrosystem level is defined as “consistencies, in the form and content of lower-order systems (…) that exist, or could exist, at the level of the subculture or the culture as a whole, along with any beliefs systems or ideology underlying such consistencies” [7]. It includes sociocultural factors such as race/ethnicity when explored from a sociocultural perspective [6,15]. Given that the definitions of “victim,” “survivor,” and “trauma” are influenced by societal imaginaries or representations [31], we have placed the impact of labeling individuals who have experienced SA as “victim” or “survivors,” and the SA event itself as “trauma,” within the macrosystem. Research has demonstrated that such labeling can have detrimental effects on their social life and their sense of self [32-35].

The chronosystem level examines “the cumulative effects of multiple sequences of developmental transitions over the life course” [6]. Research has shown that a prior history of SA and a history of other forms of violence exposure and reexposure are related to more negative psychological outcomes, which may be cumulative [6,36]. Besides, it is well-established that a history of trauma is a predictive factor for PTSD, regardless of the type of trauma experienced [18,19].

The multitude of factors and their complexity could explain the heterogeneity observed in the trajectories of individuals who have experienced SA [6,19].

### Limits of the Current Literature

Despite a growing body of literature, how these characteristics impact adaptation to trauma remains unclear [36,37] and does not provide us with the means to characterize psycho-socio-judicial trajectories [38]. Similarly, some factors remain unexplored, such as the impact of formal support on mental health outcomes [19,27,36,37]. Consequently, given that the conventional approach of predicting the development of PTSD using bivariate analyses and traditional multiple regression analysis has shown limited success, it is pertinent to investigate new predictive methods, such as AI. There are several reasons why AI might outperform conventional regression analyses: it automates the identification of hidden interactions and nonlinearities among features, enables the handling of larger and more complex data sets, and facilitates the integration of information from highly diverse sources. Additionally, it reduces the likelihood of overestimating prediction performance when dealing with numerous highly correlated independent variables [39,40]. Moreover, AI statistical algorithms represent an inductive approach, in contrast to traditional approaches that are hypothesis driven [41]. This makes AI useful for discovering unknown mechanisms [41] and uncovering relationships between data that may not be discernible through traditional statistics [39].

To date, fewer than 100 studies have used AI for predicting PTSD following a traumatic event using clinical and biological data. These studies have demonstrated a good predictive ability, with area under the curve values ranging from 75% to 98% [42,43]. However, to the best of our knowledge, only 1 study [44] has specifically included adults who experienced SA in its sample, and it reported poor predictive ability (area under the curve 64%). Moreover, other useful indicators, such as sensitivity (ie, the capacity to give a positive result when the hypothesis is verified) and specificity (ie, the capacity to give a negative result when the hypothesis is not verified), appear necessary to judge the usefulness of AI algorithm in routine clinical practice but seem to be missing in these studies, hindering the interpretation of the results and evaluation of relevance. It is therefore necessary to explore in more detail the utilization of AI to predict the onset of PTSD for people who experienced SA and to measure its usefulness for forensic services.

Furthermore, there has been minimal research conducted on the accessibility and effectiveness of support services, including psychiatric and psychological follow-ups, legal assistance, and support from associations, in Europe [38]. Thus, conducting an assessment of the functionality and quality of available resources in France is essential to describe psycho-socio-judicial trajectories accurately and to offer services that are better aligned with the needs of individuals who have experienced SA.

### Study Objectives and Aims

This study is structured into 3 major components, each fulfilling a distinct primary objective. Our first objective is to delineate the 1-year psycho-socio-judicial trajectories of individuals who have recently encountered SA and sought consultation with a forensic examiner. We hypothesize that there exist several typical psycho-socio-judicial trajectories among individuals who have recently experienced SA and sought assistance from a forensic practitioner in France. However, we also believe that a multitude of individual and contextual factors and circumstances play a crucial role in conjunction with the characterization of these trajectories [19,30].

Our second objective is to use AI to identify predictive factors for the development of PTSD. We posit that AI may be a more effective method for this prediction compared with traditional statistical methods such as multiple regression analysis [39]. A secondary goal within this primary objective is to develop an algorithm capable of screening for PTSD symptoms based on clinical criteria that are accessible during the initial forensic examination.

Our third objective is to examine the concepts of “victim” and “trauma” and enhance the understanding of SA from the perspective of individuals who have experienced it. We hypothesize that the significance and representation associated with the terms “victim” and “trauma” are either socially imposed on individuals who have experienced SA or ascribed by those involved in their recovery. However, these representations are expected to differ significantly and qualitatively from the perceptions held by the individuals directly affected by them [31,34]. Additionally, this investigation will enable us to assess the functioning and quality of available resources.

## Methods

### Overview

The research team consists of 14 professionals with expertise in forensic sciences, anthropology, psychiatry, law, criminology, and data sciences. Together, they are involved in the development and execution of our research project, named “Intelligence Artificielle, Dépistage et trajectoires psychosociojudiciaires des victimes de Violences Sexuelles” (IADViSe), which translates to “Artificial Intelligence, Screening, and Psycho-Socio-Judicial Trajectories of Sexual Violence Victims”.

### Overall Study Design

Our study is a longitudinal multicentric cohort study that uses mixed methods. Quantitative data will be gathered through repeated questionnaires administered over the course of up to a year following the SA. Qualitative data will be obtained through semistructured interviews conducted with members of the cohort, individuals who have experienced SA but are not part of the cohort, and professionals involved in their psycho-socio-judicial care. Similar to many medical and epidemiological studies [2], and given that our primary sample comprises individuals who have reported recent SA and have been referred to a medical practitioner for a formal examination, our study focuses solely on nonconsensual sexual acts that involve physical contact. This includes the legal concepts of SA (sexual contact committed by force, coercion, threats, or surprise without the clear and explicit consent of the person) and rape (SA with penetration), but excludes harassment and exhibitionism.

### Participants

#### Cohort Participants

**Figure 1:**
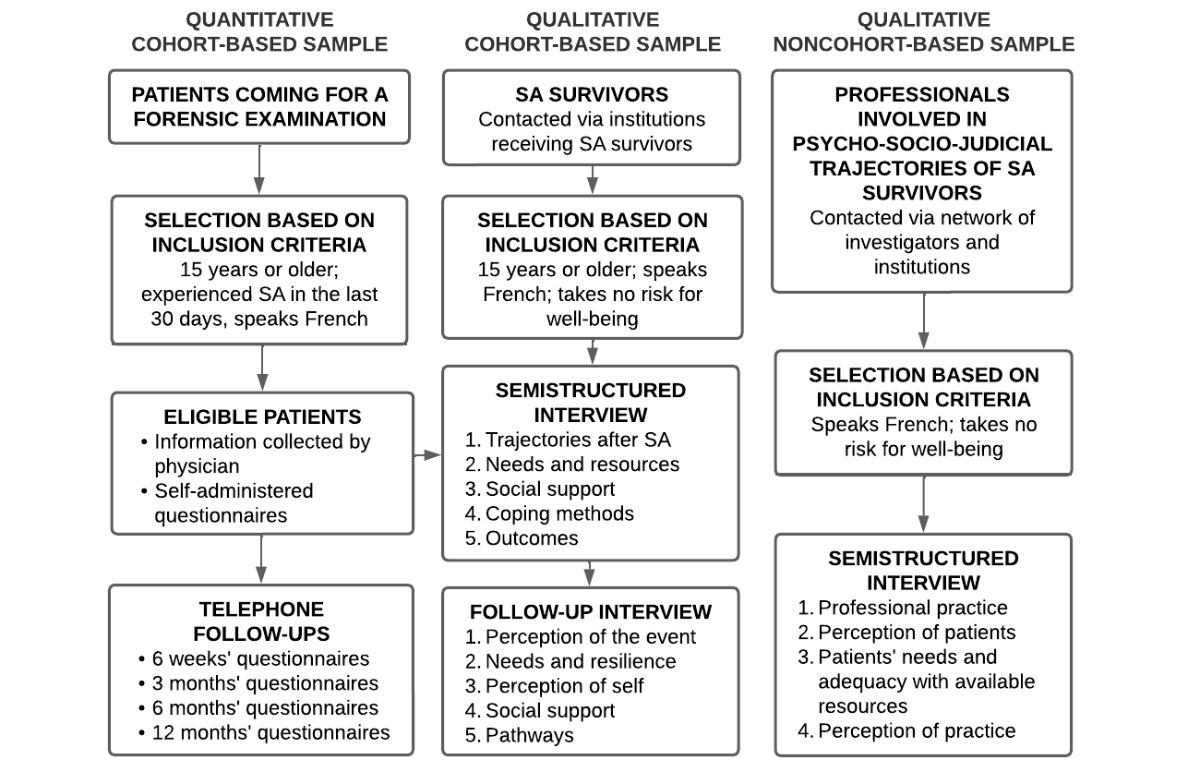
Inclusion and follow-ups stages of participants. SA: sexual assault.

An integral component of our project involves establishing a cohort of individuals who have recently experienced SA and have undergone a forensic examination, whether it was court ordered or not. This cohort will serve as the source for longitudinal quantitative and qualitative data collection (Figure 1).

#### Participant Recruitment

Participants are being recruited during their examinations for recent SA by physicians at 1 of the 5 participating forensic medical centers or medico-legal units in Île de France, which include Hotel-Dieu Hospital in Paris, Jean Verdier Hospital in Bondy, the Inter-communal Hospital Center of Créteil, the Sud Francilien Hospital in Corbeil-Essonnes, and the Versailles Hospital Center. During these examinations, physicians provide patients with consent forms that outline the purpose and methodology of the research. If patients choose to sign the consent form, their initial information and contact details are shared with one of the investigators (EF, VT, TS, or JO) responsible for follow-ups at 6 weeks, 3 months, 6 months, and 1 year after the SA. Additionally, participants are asked if they are willing to participate in interviews. If they agree, we arrange 1-3 meetings in a private setting of their choice to review the responses provided in the questionnaires and to discuss any other topics they wish to address, including their self-representations and perceptions of the SA.

#### Inclusion Criteria

We will include all individuals who undergo an SA forensic examination at 1 of the 5 forensic centers where the study is conducted, provided they consent to participate, are aged 15 or older, and have experienced SA within the last 30 days. The 30-day limit is set because one of our main objectives is to investigate the onset of PTSD, which can be formally diagnosed if symptoms persist for more than 1 month, in accordance with criterion F of the 5th edition of the Diagnostic and Statistical Manual of Mental Disorders [45].

#### Exclusion Criteria

We will exclude individuals for whom callbacks are challenging or impossible and those who do not possess sufficient proficiency in French to independently complete the questionnaires.

#### Feasibility

The potential number of participants to be included will vary depending on the clinical activity of each forensic center where the study is conducted. When considering all participating centers, there is the potential to include between 500 and 650 individuals annually. As participation in this study is entirely voluntary and offers no immediate benefits to the patients, we anticipate that not everyone will choose to participate. As a result, our recruitment period is expected to extend over a minimum of 2-3 years.

### Noncohort Participants

#### Overview

The data gathered from the cohort are supplemented with qualitative interviews involving individuals outside the cohort (Figure 1). These interviews include individuals who have experienced SA but were recruited through alternative methods, as well as professionals engaged in the psycho-socio-judicial care of SA survivors.

#### Participants Recruitment

We are recruiting noncohort participants through the networks of our investigators and by promoting our study in various organizations that assist individuals who have experienced SA, such as survivor support associations, police stations, forensic centers, regional psychotrauma centers, and more. In each organization, our designated contact person is responsible for disseminating a recruitment poster containing the investigators’ contact details, both within the organization and throughout its network where applicable. This information is shared via their website, mailing lists, in their offices, or on their social media platforms. Individuals who are interested can then reach out to the investigators to arrange a meeting. We will continue to add participants until we reach saturation.

#### Inclusion Criteria

To be eligible for participation, individuals must be aged 15 or older, have a history of experiencing SA or work closely with individuals who have experienced SA, provide their consent to participate in the research, and possess sufficient proficiency in French to be interviewed without requiring a translator.

#### Exclusion Criteria

Individuals who are unable to arrange a face-to-face or video meeting in a location that ensures their privacy and does not jeopardize their well-being will be excluded from participation.

### Ethical Considerations

All procedures, including the process of obtaining informed consent, underwent a thorough ethical review and received approval from the Research Ethics Committee of Paris Cité University (institutional review board approval number: 00012022-14, date: April 5, 2022). All experiments and methodologies are conducted in strict accordance with the principles outlined in the Declaration of Helsinki**.**

Informed consent is obtained from all study participants through the signing of a printed consent form. This form explicitly outlines the study’s objectives, methodology, and associated risks. The consent process is facilitated either by the physician during the forensic examination or by one of the investigators at the commencement of the first interview for noncohort participants. Physicians and investigators are readily available to address any questions or concerns that participants may have regarding the study. Furthermore, participants are informed of their right to withdraw from the study at any point in time.

Participants may be experiencing latent psychological distress, with or without PTSD, either in the usual aftermath of the events or potentially triggered during the forensic examination or telephone contacts and interviews. However, most of these risks are not attributable to the research project itself. The security of participants during the forensic examination is the responsibility of the medical examiner, who follows the safety guidelines required by their department. To ensure the security of participants during and between follow-ups, investigators, all of whom are trained in dealing with people who have been exposed to SA, remind participants that some questions may be triggering, emphasize their right to stop their participation at any moment and ensure that participants are not in distress at any time during the call. As they are already part of an identified medical and psychological care system, participants will be asked to contact the forensic medical center where they were received if necessary. A list of professionals and resources is also provided to all participants for information and guidance. Participation is voluntary and does not include compensation, but it can be beneficial to participants who need to discuss their experiences.

Each participant is assigned an inclusion number to anonymize the database entries and questionnaires. Physicians or investigators record identifying and contact information in tables outside the database, allowing each participant to be linked to their inclusion number and recontacted. The audio of the interviews is recorded, transcribed, and anonymized with the same embedding code where applicable. All documents allowing the identification of the participants are securely stored and located in each participation center and will be destroyed as soon as the data collection is completed. After the data collection is completed and the database is appropriately anonymized and cleaned, the data and all necessary materials for their use will be uploaded to a public archive, such as HAL (Hyper Article en Ligne, which translates to Online Hyper Article), under a CC (creative commons) license, in compliance with the FAIR (Findable, Accessible, Interoperable, Reusable) principles and GDPR (General Data Protection Regulation) regulations.

### Quantitative Measures

Quantitative measures at each time point are presented in Table 1. Initial data are collected during the forensic examination. The physician completes a questionnaire (see Multimedia Appendix 1) based on information that forensic practitioners routinely collect for their report to the criminal justice system. The questionnaire is adapted from a previously constructed questionnaire [46], which is still in use today by forensic practitioners in the Department of Forensic Medicine at the Jean Verdier Hospital (Bondy, France). Participants are also asked to complete 2 self-assessment questionnaires on peritraumatic dissociation and distress. Investigators contact the participants again at 6 weeks, 3 months, 6 months, and 1 year after the SA so that they can answer follow-up questionnaires regarding their health, support, and the status of their judicial proceedings. These follow-up sessions do not exceed 40 minutes.

**Table 1:** Characteristics and outcome measures and time points at which they are collected.

Variable category and variables		Measure	Timeline				
			Baseline (30 days maximum)	6 weeks	3 months	6 months	1 year
**Individual characteristics**							
	Sociodemographic variables^a^	Bespoke measures	✓	✓			
	Medical and psychiatric history and treatment	Bespoke measures	✓				
	Coping	Brief-COPE (French version)		✓	✓	✓	✓
	Substance misuse	HSI^b^, CAGE^c^, CAST^d^ (French versions), and ECAB^e^		✓	✓	✓	✓
**Assault characteristics**							
	Aggressor and aggression information^f^	Bespoke measures	✓				
**Microsystem**							
	Received social support	RNAE-22^g^		✓	✓	✓	✓
**Exosystem (formal support)**							
	Use of services	Bespoke measures		✓	✓	✓	✓
	Quality of contact	Bespoke measures	✓	✓	✓	✓	✓
	Perceived negative support	From Campbell and Raja [30]		✓	✓	✓	✓
	Secondary victimization	Bespoke measures		✓	✓	✓	✓
**Chronosystem**							
	Childhood history of violence and exposition to domestic violence	Bespoke measures	✓				
	Prior assault history	Bespoke measures	✓				
	Sexual violence reexposure	Bespoke measures		✓	✓	✓	✓
**Functional outcomes**							
	Peritraumatic distress	PDI^h^ (French version)	✓				
	Peritraumatic dissociation	PDEQ^i^ (French version)	✓				
	Physical and psychological state at examination	Bespoke measures	✓				
	Posttraumatic stress disorder symptoms	PCL-5^j^ (French version)		✓	✓	✓	✓
	Quality of life	SF-12v2^k^ (French version)		✓	✓	✓	✓
	Impact on daily life	WHODAS 2.0^l^ (French version)		✓	✓	✓	✓

^a^Sociodemographic variables are age, gender identity, marital status, employment status, level of education, sexual identity, and race/ethnic identity.

^b^HSI: Heaviness of Smoking Index.

^c^CAGE: Cut Annoyed Guilty Eye-Opener.

^d^CAST: Cannabis Abuse Screening Test.

^e^ECAB: Echelle Cognitive d’Attachement au Benzodiazépine (Benzodiazepine Cognitive Attachment Scale).

^f^Aggressor and aggression information includes the number of aggressors, their gender, age, their relation to the impacted individual, alcohol or substance use from the aggressor and the impacted individual, the impacted individual’s physical reaction of defense, impacted individual’s amnesia, suspicion of chemical submission, the location of the sexual assault, types of sexual assault, and associated violence.

^g^RNAE-22: Questionnaire sur les réactions nuisibles et aidantes de l’entourage lors d’un dévoilement de violences à caractère sexuel – Version francophone adaptée

^h^PDI: Peritraumatic Distress Inventory.

^i^PDEQ: Peritraumatic Dissociative Experience Questionnaire.

^j^PCL-5: Posttraumatic Stress Disorder Checklist for DSM-5.

^k^SF-12v2: Short Form 12-item version 2 Health Survey.

^l^WHODAS 2.0: World Health Organization Disability Assessment Schedule 2.0.

### Individual Factors

#### Sociodemographic Variables

Sociodemographic data are first collected during the initial examination. These include participants’ age (in years) and gender (1=female, 2=male, and 3=other), the name of the forensic center in which the examination took place, the anonymized identity of the practitioner, and the time between the assault and the examination (in hours). Complementary sociodemographic variables are collected during follow-ups. These include marital status (0=never married, 1=married, 2=separated, 3=divorced, 4=widowed, and 5=cohabitation) and employment status (1=employee, 2=independent worker, 3=unpaid worker, 4=student, 5=housewife/husband, 6=retired, 7=unemployed due to health reasons, 8=unemployed for another reason, and 9=other), which are based on sociodemographic information measured in the World Health Organization Disability Assessment Schedule 2.0 (WHODAS 2.0) questionnaire [47]. The level of education (from 0=no diploma to 10=master’s degree; and 11=other) is based on the sociodemographic questions in the 2019 Health Barometer Study [48]. Sexual identity (1=heterosexual, 2=gay or lesbian, 3=bisexual or pansexual, and 4=other) is based on an item recommended by the University of California (UCLA) Sexual Minority Assessment Research Team (SMART) team [49]. We created an item to collect information about gender diversity identification (0=no and 1=yes). The last sociodemographic variable collected is ethnicity/race (1=White, 2=Black, 3=Middle Eastern, 4=Hispanic/Latinx, 5=Asian, and 6=other) and is based on the recommendation from Public Health Ontario [50] and adapted to a French context.

#### Medical and Psychiatric History and Treatments

We will collect data recorded by the practitioners during the initial examination, including a history of serious or chronic diseases, personal and familial psychiatric history, and current medical treatments that could impact psychological well-being (eg, mood stabilizers, anxiolytic, hypnotic, antidepressant, neuroleptic, analgesic drugs).

#### Coping

Coping strategies are measured at each follow-up (6 weeks, 3 months, 6 months, and 1 year) using the French version [51] of the Brief-COPE [52]. This 28-item instrument measures the frequency with which an individual uses different positive and maladaptive coping strategies on a 4-point Likert scale ranging from 0=not at all to 3=a lot. The Brief-COPE and the COPE inventory are widely used tools for assessing coping strategies and have been translated and validated in at least seven languages, facilitating international replication and comparisons [51]. The French version of this instrument demonstrates good psychometric properties [51].

#### Follow-Up Substance Use

Tobacco, alcohol, delta-9-tetrahydrocannabinol, and benzodiazepine use since the assault is assessed at each follow-up (6 weeks, 3 months, 6 months, and 1 year) by inquiring whether participants use each substance and whether their consumption has increased or decreased since the assault [53]. If participants report using these substances, follow-up questionnaires are administered to assess whether their use of each substance is problematic. This assessment is performed using the 2-item simplified Fagerström test [54], a French version of the Heaviness of Smoking Index (HSI) [55], the French version of the Cut Annoyed Guilty Eye-Opener (CAGE) test (Diminuer Entourage Trop Alcool; DETA) [56], the French version of the Cannabis Abuse Screening Test (CAST) [57], and the Echelle Cognitive d’Attachement au Benzodiazépine (Benzodiazepine Cognitive Attachment Scale; ECAB) [58]. All of these instruments are validated and recommended by the French High Authority for Health.

#### Assault Characteristics

Standard consultation information about the assailant (ie, number, gender, age, relation to the impacted individual, and alcohol or substance use), the aggression (ie, location of the assault, types of violence, and context of the assault), and the impacted individual (ie, physical defense reaction, amnesia, chemical submission, and substance or alcohol use) is collected during the initial forensic examination as part of the physicians’ routine screening.

### Microsystem (Informal Support System)

We will assess social support during follow-ups at 6 weeks, 3 months, 6 months, and 1 year using the *Questionnaire sur les réactions nuisibles et aidantes de l’entourage lors d’un dévoilement de violences à caractère sexuel – Version francophone adaptée* (RNAE-22) [59]. This questionnaire is a French version of the Social Reaction Questionnaire–Short (SRQ-S) [60] adapted to be applicable to all genders. This instrument measures the frequency of positive and negative social reactions to an SA disclosure on a 5-point Likert scale ranging from 0=never to 4=always. Items are split into 3 subscales describing the behavior of people receiving the disclosure: Turning against, Unsupportive acknowledgment, and Positive support, which have been validated in their original [60] and French versions [59].

### Exosystem (Formal Support System)

#### Use of Judicial, Medical, Mental Health, and Community Services

The use of different formal services (judicial, medical, psychological, and community) is measured at each follow-up (6 weeks, 3 months, 6 months, and 1 year) with questions built by the research team based on empirical findings and the perspective of care providers and legal professionals, such as “Have you filed a complaint with the police department?” We inquire about the evolution of medical, psychological, and community care, as well as the progress of the legal procedure at each follow-up.

#### Quality of Contact With the Exosystem

The quality of the contact between the physician and the patient is initially measured during the initial examination, where the physician is asked to rate it on a scale ranging from 0=very bad to 6=excellent. Participants are subsequently asked to evaluate the quality of their contact with each formal system during follow-ups, including their interactions with the medical system.

#### Negative Perceived Social Support by the Exosystem

The participant’s negative perceived support from each formal service they used is assessed at each follow-up (6 weeks, 3 months, 6 months, and 1 year) using 5 items adapted from a study by Campbell and Raja [30]. These items examine various negative feelings the participant may have experienced after their interaction with each system, including guilt, depression, anxiety, distrust, and reluctance to seek further help.

#### Secondary Victimization

We will assess secondary victimization at each follow-up (6 weeks, 3 months, 6 months, and 1 year) by inquiring whether participants have experienced any guilt-inducing or unhelpful behaviors from justice system officials. Examples of such behaviors are “being pressured to withdraw your complaint” or “questioning your words.” The list of these secondary victimization behaviors was developed by the research team, drawing from empirical findings and insights from legal system professionals.

### Chronosystem

#### History of Violence During Childhood

We will measure participants’ history of psychological, physical, and sexual violence exposure (by the same aggressor or by another); history of intrafamilial psychological, physical, and sexual violence exposure; and instances of witnessing domestic violence in their childhood. These questions are administered during the initial forensic examination.

#### Sexual Violence Reexposure

We will assess instances of physical, psychological, and sexual violence reexposure at each follow-up point (6 weeks, 3 months, 6 months, and 1 year) by inquiring whether the participant has experienced any of these forms of violence, by the same assailant or another, since the initial assault.

### Functional Outcomes

#### Peritraumatic Distress

Peritraumatic distress is measured during the forensic examination using the French version of the Peritraumatic Distress Inventory (PDI) [61]. This instrument measures the frequency with which participants experienced various negative feelings, such as “feeling helpless” or “feeling like dying” during or directly after the assault, on a 5-point Likert scale ranging from 0=not at all to 4=extremely, and shows good psychometric properties [61].

#### Peritraumatic Dissociation

Peritraumatic dissociation is measured during the forensic examination using the French version of the Peritraumatic Dissociative Experience Questionnaire (PDEQ) [62]. This instrument measures 10 symptoms of dissociation during and immediately after an event on a 5-point Likert scale ranging from 0=not at all to 4=extremely and shows good psychometric properties [62].

#### State at the Time of Examination

We will gather information on physical complaints and injuries, the participant’s psychological state, and the duration of “total or temporary work incapacity” (a legal term used in France to evaluate the severity of the SA) as reported by the physician during the consultation.

#### PTSD Symptoms

We will assess PTSD symptomatology at each follow-up (6 weeks, 3 months, 6 months, and 1 year) using the French version of the Posttraumatic Stress Disorder Checklist for DSM-5 (PCL-5), which has been validated both in French and in its original version [63]. This 20-item instrument evaluates the PTSD symptoms experienced by participants during the past month. It uses a 5-point Likert scale ranging from 0=not at all to 4=extremely. A total score of 31-33 or higher suggests that the participant may meet the criteria for PTSD and could benefit from PTSD treatment.

#### Quality of Life

The Short Form 12-item version 2 Health Survey (SF-12v2) [64] will be used to assess the physical, mental, and social quality of life during the past month. This survey is administered at each follow-up (6 weeks, 3 months, 6 months, and 1 year). Each item contributes to the scores of the Physical and Mental Component Summary. Both indexes are converted to a scale from 0 to 100, and the mean score of the sample can be compared with the national norm (where the national mean is 50 with an SD of 10).

#### Impact on Daily Life

We will assess the impact of the assault on daily life, including personal activities, social activities, and work, at each follow-up (6 weeks, 3 months, 6 months, 1 year) using the WHODAS 2.0 questionnaire [47]. This 12-item instrument measures the extent of these impacts on a Likert scale ranging from 0=none to 4=extremely.

### Qualitative Interviews

Semidirective interviews are conducted with both cohort participants and individuals who have experienced SA in the past but are not part of the cohort (see Multimedia Appendix 2). These interviews will complement our quantitative data collection. The initial 1-hour semidirective interview covers topics such as the individual’s perception of the event, formal and informal support, coping methods, and the event’s impacts. After establishing a good rapport between the participant and the interviewer, additional interviews (second or third) can delve deeper into topics discussed in previous sessions. These interviews explore the participant’s value system, their integration of the event into their life course, and their perceptions of themselves and the SA in relation to the concepts of “trauma” and “victim.”

Other semidirective interviews are conducted with professionals involved in the pathways of people who have experienced SA (see Multimedia Appendix 3). These interviews cover various aspects: first, professionals discuss their practice and the needs of their patients or clients; second, they share their perceptions of the help they provide, the adequacy of available resources, and the needs of SA survivors; finally, they provide insights into their definitions of the notions of “trauma” and “victim,” and discuss the utility of these terms in their field and for SA survivors.

### Statistical Analysis Planned: Quantitative Analysis

Descriptive analyses of participant characteristics will be conducted. The total population initially included and the follow-up cohort will be compared to understand the differences between patients who abandon their participation and those who complete their follow-up.

Bivariate and multivariate analyses, including trajectory analyses, will be conducted to examine the associations between each variable and mental, physical, social, and judicial outcomes at 6 weeks, 3 months, 6 months, and 1 year after the SA. Results regarding the presence or absence of PTSD will serve as the basis for our predictive analyses.

We will conduct predictive analyses using multiple prediction algorithms, such as meta-learners and support vector machines, to predict PTSD at the initial examination. The testing phase will be carried out in a subsequent study to validate the algorithms using a subset of our cohort. Initially, we will select relevant variables automatically, either through feature selection or manual selection based on the results of multivariate analyses. We will assess the internal validity of the prediction model through cross-validation. To quantify the performance of the algorithms, we will use metrics such as precision (accuracy and area under the precision-recall curve), sensitivity, specificity, positive predictive value, and negative predictive value. Additionally, we will explore tools for visual explanation, feature relevance explanation, and explanation by example [65] to provide elements of explainability.

### Qualitative Analysis and Data Integration

Interviews conducted with individuals who have recently experienced SA, with those who have experienced SA in the past, and with professionals will be transcribed before performing coding and thematic analysis. The results of interviews with recent SA survivors included in the cohort will be integrated with the quantitative data, following best practices for mixed methods research [66]. This integration will enable us to add or enhance existing variables to be used in constructing our predictive model for PTSD using AI. Furthermore, it will provide more comprehensive information on the 1-year psycho-socio-judicial trajectories of participants, allowing for a more precise characterization of these trajectories, particularly concerning the judicial aspect and the role of the exosystem (formal support system), which have not been extensively analyzed in quantitative literature.

The data concerning the perceptions of the significance and utility of the concepts of “trauma” and “victims” will be analyzed separately to gain deeper insights into the representations and definitions of SA and individuals who have experienced it. This data will be conducted from the perspectives of recent SA survivors (cohort participants), those who have experienced SA in the past, and professionals who provide assistance to individuals impacted by SA (noncohort participants).

## Results

Data collection commenced in September 2022 and is anticipated to conclude by 2026. Data analysis will commence before the conclusion of data collection, with several articles expected to be prepared and submitted for publication between 2023 and 2026.

## Discussion

### Anticipated Findings

Our first 2 study objectives involve delineating the psycho-socio-judicial trajectories over 1 year of individuals who undergo forensic examinations after a recent SA and using AI for early detection of those at risk of developing PTSD. Third, using mixed methods and conducting interviews with both individuals who have recently experienced SA and those with nonrecent experiences, in addition to professionals engaged in their care, we aim to assess the functionality and attributes of the available resources in France. This approach will contribute to a deeper understanding of SA, its integration into the life course, and the perceptions of “victim” and “trauma” as perceived by those who have experienced it.

### Limitations

Our study has certain limitations. In France, the majority of forensic centers require a court order to admit patients, and this order is typically granted once the alleged impacted individual files charges. However, only 7%-8% of people who experience SA choose to press charges [67], and it is possible that this small percentage may not accurately represent the experiences of all individuals who have been subjected to such violence. As a result, individuals who undergo a forensic examination following SA represent a specific subset of individuals and types of assaults. In our study, the forensic center at Jean Verdier Hospital is the only one that does not require patients to have a court order for examination. Consequently, only a small portion of our cohort sample will consist of individuals who have not pressed charges. This limitation could potentially affect the representativeness of our sample.

Furthermore, our specific age and temporal criteria necessarily exclude individuals under the age of 15 years and those whose experience of SA occurred more than 30 days ago. This exclusion limits the participation of individuals who have experienced certain types of sexual violence, such as incest or child sexual abuse. This criterion is necessary to study the onset of PTSD, which typically occurs 1 month after the trauma according to DSM-5 criterion F [45]. Additionally, it facilitates the inclusion of participants, as individuals aged 15 years and older can provide consent for matters related to their sexual health. However, this criterion introduces a potential selection bias, as it excludes individuals under the age of 15 years.

There is also a potential for interviewer bias in the qualitative part of the study. However, several measures have been put in place to minimize interviewer bias. These include using a standardized interview guide for all participants, predesigning codes based on the framework used, ensuring the interviewer is trained in qualitative interviews with crime offenders and impacted individuals, adopting an inductive approach, maintaining long-term monitoring of participants to enable reflexivity on practice, and allowing for reformulation of hypotheses and material as the fieldwork progresses. Additionally, inclusions are based on specific criteria and are not made by the interviewer but by different forensic practitioners, further enhancing reflexivity and limiting this bias.

Lastly, it is important to note that part of our study involves the use of AI, which can be more complex to implement and manipulate compared with standard statistical methods [39]. Previous research has highlighted several challenges associated with the integration of AI into studies on psychiatric disorders and its application in clinical settings. These challenges include issues related to model validation; computational resources; data quality; multimodal data; nonstationary data distributions; the heterogeneity of clinical diagnoses; the interpretability of AI models; conservative health care funding practices; and ethical concerns such as data privacy, anonymity, and the potential impact of predictive models on individuals [68].

Despite these limitations, this study plays a crucial role in filling knowledge gaps concerning the psycho-socio-judicial consequences of recent SA, the development and progression of PTSD symptoms, and the requirements of individuals who have experienced SA.

### Strengths and Expected Implications

On the quantitative aspect of the project, the systematic data collection process across multiple forensic medicine centers, each with its own unique practices and recruitment patterns, will offer unparalleled insights into individuals seeking consultation for SA across various dimensions, as well as into the practices of professionals involved in these cases. This will enable us to monitor the emergence of significant psychological disorders, including PTSD, and assess their consequences on the life trajectories and functional outcomes of the participants. Furthermore, our quantitative design uses well-established and commonly used scales to assess psychological trauma and the functional consequences of trauma, facilitating comparisons with other national and international research.

Another significant quantitative advantage of our study is its emphasis on predicting the development of PTSD through the application of AI. Indeed, the data gathered during the initial forensic examinations will serve as the foundation for developing initial AI algorithms to identify individuals at risk of developing PTSD. This study will serve as the foundation for a second study, which will be integrated into the project, aimed at investigating the contribution of AI in the early detection of PTSD, as well as the reception and integration of this tool by medico-social services. We hope to develop tools to better guide individuals who have experienced SA from their initial forensic examination, particularly in terms of psychotrauma. This could potentially yield novel findings, as there is promising evidence that AI can be of great assistance in medical services [42,43], although it has not, to our knowledge, been tested in the context of forensic examinations following SA.

Regarding the qualitative aspect of the project, it stands as one of the few endeavors exploring the characteristics and challenges of French infrastructures and the requirements of individuals who have experienced SA in France. This will enable us to map the existing, accessible, and deficient resources within their paths. It is also uncommon in medical and public health research to longitudinally follow individuals who have experienced SA while placing them at the center of the study, enabling us to redefine the representations of their experiences. We aspire to gain a deeper understanding of the individuals seeking help after experiencing SA, their perceptions, and the appropriateness of the support services available to them across various contexts and timeframes. By comprehensively examining how individuals who have experienced SA perceive and incorporate this event into their life course, we can gain valuable insights into their needs and work toward enhancing their support and care. This involves considering the entire medico-social infrastructure in France and its capabilities to address these needs effectively.

### Conclusions

This project will allow us to collect numerous data never before collected over such long periods in medical sciences not only in France but also globally. The wealth of data collected is expected to yield unprecedented insights and findings. First, our project will advance research on the impact of risk and resilience factors on trauma adaptation and the characterization of trajectories using innovative methods and extensive quantitative and qualitative longitudinal data. Second, it is a first step toward a functioning AI tool for early diagnosis of PTSD, which will enable us to prevent the chronicization of PTSD and provide better guidance to individuals during their first forensic examination. Third, this study will help bridge the existing gap in the literature regarding the accessibility and effectiveness of support services for individuals who have experienced SA in Europe by comprehensively mapping the currently available resources while also identifying areas where resources may be lacking. This assessment will be based on the needs and perspectives of individuals who have experienced SA, considering the impact of the events on their lives. Furthermore, this innovative research, which encompasses the entire psycho-socio-judicial chain, places a central emphasis on the perspectives of those who have experienced SA. This approach will enable the formulation of novel recommendations aimed at improving the quality of care and support provided at all levels of the system, commencing with the initial forensic examination.

We hope our approach may fuel further research to investigate psycho-socio-judicial trajectories via the creation of cohorts of people who experienced SA in other settings to have a more representative view of all people who have been sexually assaulted. Future research should also continue to focus on the use of AI to help medical examiners refer their patients toward more adapted services by testing the algorithms that will be developed in this project on several samples with different sociodemographic and trauma characteristics.

## Appendix

Multimedia Appendix 1 Forensic examination questionnaire of the IADViSe study.

Multimedia Appendix 2 Semi-directive interview guide for participants who experienced sexual assault.

Multimedia Appendix 3 Semi-directive interview guide with professionals.

## Abbreviations

AI: artificial intelligence
CAGE: Cut Annoyed Guilty Eye-Opener
CAST: Cannabis Abuse Screening Test
CC: creative commons
DSM-5: Diagnostic and Statistical Manual of Mental Disorders, Fifth edition
ECAB: Echelle Cognitive d’Attachement au Benzodiazépine (Benzodiazepine Cognitive Attachment Scale)
FAIR: Findable, Accessible, Interoperable, Reusable
GDPR: General Data Protection Regulation
HAL: Hyper Article en Ligne, which translates to Online Hyper Article
HIS: Heaviness of Smoking Index
IADViSe: Intelligence Artificielle, Dépistage et trajectoires psychosociojudiciaires des victimes de Violences Sexuelles (Artificial Intelligence, Screening, and Psycho-Socio-Judicial Trajectories of Sexual Violence Victims)
MRA: multiple regression analysis
PDEQ: Peritraumatic Dissociative Experience Questionnaire
PDI: Peritraumatic Distress Inventory
PTSD: posttraumatic stress disorder
SA: sexual assault
SF-12v2: Short Form 12-item version 2 Health Survey
SRS-Q: Social Reaction Questionnaire–Short
WHODAS 2.0: World Health Organization Disability Assessment Schedule 2.0

## Availability of Data and Materials

Data and materials will be available on an open-access archiving platform such as the UK Data Archive once the data collection is completed.
