# Supplementary material for: Identification of Psycho-Socio-Judicial Trajectories and Factors Associated With Posttraumatic Stress Disorder in People Over 15 Years of Age Who Recently Reported Sexual Assault to a Forensic Medical Center: Protocol for a Multicentric Prospective Study Using Mixed Methods and Artificial Intelligence
Source: JMIR Res Protoc. 2023 Oct 16;12:e46652. doi: 10.2196/46652 (PMC10616743; doi:10.2196/46652)
Supplement: Multimedia Appendix 1 [file resprot_v12i1e46652_app1.pdf]

0. Identifying data (for investigators only)

Patient identity :
Name:
First Name :
Date of birth :
Phone :
Email :

Examination date :
Examination time :
Forensic center:
Forensic examiner:

✄--

Participation number :
Examiner number :

Legal complaint : ☐ yes ☐ no

Time between the events and the examination **:** …………… days or …………… hours

Examination type : ☐ Initial examination ☐ Follow-up examination **If follow-up, time between the initial exam and the follow-up :** ……… days

1. Information on the victim

Sex : ☐ M ☐ F ☐ other **Age :** ……………

History of serious or chronic illness (other than psychiatric) : ☐ none ☐ yes, *specify :*

Personal psychiatric history : ☐ none ☐ depression ☐ suicide attempt(s) ☐ psychotic disorders ☐ anxiety disorder ☐ bipolar disorder ☐ other, *specify :*

Current mental health care : ☐ none ☐ psychiatrist ☐ psychologist ☐ physician

Familial psychiatric history : ☐ none ☐ yes, *specify :*
(Grandparents, parents, siblings)

History of abuse (other than childhood intrafamilial abuse) :
from the same assailant : ☐ none ☐ psychological ☐ physical ☐ sexual
from another assailant : ☐ none ☐ psychological ☐ physical ☐ sexual

Childhood intrafamilial abuse history : ☐ none ☐ neglect ☐ psychological ☐ physical ☐ sexual

If yes, assailant : ☐ brother ☐ sister ☐ father ☐ mother ☐ stepfather ☐ stepmother ☐ uncle ☐ aunt ☐ grandfather ☐ grandmother

Witness of interpersonal violence : ☐ no ☐ yes

Current medication : ☐ none ☐ anxiolytic ☐ hypnotic ☐ antidepressant ☐ thymoregulator ☐ neuroleptic ☐ analgesic ☐ other, *specify :*

2. Information on the assailant

Number : ☐ unique ☐ multiple ☐ unknown

Sex : ☐ M ☐ F ☐ unknown **Age :** ……………

Relation to the victim : ☐ stranger ☐ intimate partner ☐ parent ☐ sibling ☐ other family member ☐ ex-intimate partner ☐ friend ☐ schoolmate ☐ manager / supervisor ☐ colleague ☐ met online ☐ authority relationship ☐ other known person ☐ other ☐ unknown

Alcohol or psychoactive substance use by the assailant during the event : ☐ unknown ☐ no ☐ yes, *specify :*

3. Characteristics of the agression

Place : ☐ victim's residence ☐ assailant's residence ☐ shared residence ☐ other private place ☐ workplace ☐ car ☐ other place ☐ unknown ☐ public place, public transport, street ☐ school or college

Specific context : ☐ total amnesia ☐ partial amnesia ☐ suspicion of chemical submission ☐ none

Alcohol or psychoactive substance used by the victim during the event : ☐ unknown ☐ no ☐ yes, *specify :*

Type of sexual violence : ☐ molestation ☐ digital anal penetration ☐ digital vaginal penetration ☐ penile oral penetration ☐ penile anal penetration ☐ penile vaginal penetration ☐ object penetration ☐ sexual harassment ☐ cunnilingus ☐ sexual mutilation ☐ forced prostitution ☐ unknown
Number : ☐ unique ☐ repeated

Associated violence :
Insults ☐ yes ☐ no ☐ unknown
Threat ☐ none ☐ verbal (death, violence) ☐ with a stabbing weapon ☐ with a firearm ☐ with a sharp object ☐ other ☐ unknown

Physical violence ☐ none ☐ slapped ☐ punched ☐ kicked ☐ hit with an object
☐ stabbed ☐ pushed to the ground ☐ strangulation ☐ held ☐ crushed
☐ bitten ☐ hair pulled ☐ other ☐ unknown

Sequestration ☐ yes ☐ no ☐ unknown

Physical defence reaction : ☐ yes ☐ no ☐ unknown

4. Peritraumatic distress and dissociation

IMPORTANT : Specific questions/information to ask during the examination; may not be found in the file systematically.
Ideally, ask the questions directly to the victim; by default, check off each item yourself according to the information gathered during the interview.

Peritraumatic distress : *Inventory of feelings or emotions experienced by the person **during** or **immediately after the assault***

1) Did you feel **helpless** ?
☐ yes ☐ no ☐ Unknown

2) Did you feel **sad** ?
☐ yes ☐ no ☐ Unknown

3) Did you feel **angry** or **frustrated** ?
☐ yes ☐ no ☐ Unknown

4) Did you **fear for your security** ?
☐ yes ☐ no ☐ Unknown

5) Did you feel **guilty** ?
☐ yes ☐ no ☐ Unknown

6) Did you feel **ashamed** of your own reactions ?
☐ yes ☐ no ☐ Unknown

7) Have you been **worried about other** ?
☐ yes ☐ no ☐ Unknown

8) Did you feel like you were **losing control** ?
☐ yes ☐ no ☐ Unknown

9) Have you had an urge to **urinate or defecate** ?
☐ yes ☐ no ☐ Unknown

10) Were you **horrified** by what occurred to you ?
☐ yes ☐ no ☐ Unknown

12) Did you think you might **die**?
☐ yes ☐ no ☐ Unknown

11) Did you feel like you might **faint** ?
☐ yes ☐ no ☐ Unknown

13) Have you had any **physical reaction to stress** such as sweating, trembling or palpitations ? ☐ yes ☐ no ☐ Unknown

Peritraumatic dissociation : ☐ yes ☐ no ☐ Unknown
Check « yes » if the person had at least one dissociative experience or reaction during or immediately after the assault (e.g., altered perception of time, place, self, or reality; impression of having been on "automatic pilot")

5. Forensic examination

Physical complaint : ☐ none ☐ fatigue ☐ pelvic symptoms ☐ pelvic pain
☐ head/face pain ☐ neck pain ☐ truck pain ☐ upper limbs pain
☐ lower limbs pain ☐ other, *specify :*

Examination performed : ☐ physical ☐ genital ☐ anal ☐ none

Reason if physical examination not performed: ☐ not indicated ☐ refused
Reason if pelvis examination not performed: ☐ not indicated ☐ refused

Observed physical lesion : ☐ none ☐ yes ☐ NA
(in relation to the agression)

If lesion, specify its nature : ☐ ecchymosis/hematoma ☐ wound ☐ scar ☐ osteoarticular damage

Observed genital lesion : ☐ none ☐ yes ☐ NA
(in relation to the agression)

Observed anal lesion : ☐ none ☐ yes ☐ NA
(in relation to the agression)

Psychological symptoms : ☐ flashback ☐ ruminations ☐ anxiety ☐ affective indifference ☐ sadness
☐ dark thoughts ☐ suicida thoughts ☐ peritraumatic amnesia ☐ avoidance ☐ withdrawal
☐ irritability ☐ sleep problems ☐ appetite problems ☐ desire for sanction ☐ injustice
☐ anger ☐ culpability ☐ shame ☐ psychotic elements ☐ agitation
☐ fear ☐ concentration problems ☐ hypervigilance ☐ derealisation/depersonalisation
☐ none

Length of ITT : …………… days ☐ not evaluated ☐ not asked

Intensity of functional impairment (between 0 and 6) : ……………
(0 : no impairment ; 6 : very important impairment)

Quality of contact between the examiner and the victim (between 0 and 6) : ……………
(0 : very bad/no contact ; 6 : excellent contact)

Oral information delivered to the victim : ☐ yes ☐ no
Victim's consent to participate in the study : ☐ yes ☐ no
