# Supplementary material for: Identification of Psycho-Socio-Judicial Trajectories and Factors Associated With Posttraumatic Stress Disorder in People Over 15 Years of Age Who Recently Reported Sexual Assault to a Forensic Medical Center: Protocol for a Multicentric Prospective Study Using Mixed Methods and Artificial Intelligence
Source: JMIR Res Protoc. 2023 Oct 16;12:e46652. doi: 10.2196/46652 (PMC10616743; doi:10.2196/46652)
Supplement: Multimedia Appendix 2 [file resprot_v12i1e46652_app2.pdf]

Perception of the assault and integration	**Today, how do you perceive or describe what you have experienced?**<br>**Subquestion 1** : When and how did you put the words sexual assault on what you experienced?<br>**Subquestion 2** : How would you describe what you experienced?<br>**Subquestion 3** : In your opinion, did the way the assault occurred impact how you processed or understood it?<br>**Subquestion 4** : How do you make sense of what happened?
Individual factors : resilience, needs	**What do you need today ? What's important to you ?**<br>**Subquestion 1**: What motivates you today?<br>**Subquestion 2** : What makes you feel good ? How do you concretely deal with this event?<br>**Subquestion 3** : Have these values or motivations changed since the event?
Outcomes, perception of self and others	**In relation to other significant events, how would you rate this event?**<br>**Subquestion 1** : Impacts on daily life / relationships / professional life / physical health / mental health / sexual health.<br>**Subquestion 2** : How do you see yourself today ?<br>**Subquestion 3** : Do you feel like this way of seeing yourself is consistent with how others see you?
Microsystem factors : social support	**Could you describe the reactions of those around you when you talked about what you had experienced and the support they gave you?**<br>**Subquestion 1** : Who did you talk to about what you experienced and why?<br>**Subquestion 2** : What did you think of the support they gave you?<br>**Subquestion 3 :** How did this support help or hinder your recovery?
Psycho-socio-judicial pathway	**Could you describe the contacts you have had with the judicial system and the various medical, psychological and community services ?**<br>**Subquestion 1 (judicial system) :** Can you describe your journey from the moment you decided to file a complaint (decision to file a complaint / how you were received / your feelings, etc.) ?<br>**Subquestion 2 (medical system)** : What kind of help did you receive / how accessible was it / how beneficial ?<br>**Subquestion 3 (psychosocial system)** : What kind of help did you receive / how accessible was it / how beneficial ?<br>**Subquestion 4 :** Are the resources you have accessed in line with the needs you have or had at the time of consulting them? Why or why not? What would you have preferred?
Final questions	Today, how do you see the continuation of your life-course?<br>Do you have anything to add?<br>What did you think of this interview?<br>Why did you decide to participate in this interview today?
