# Supplementary material for: Identification of Psycho-Socio-Judicial Trajectories and Factors Associated With Posttraumatic Stress Disorder in People Over 15 Years of Age Who Recently Reported Sexual Assault to a Forensic Medical Center: Protocol for a Multicentric Prospective Study Using Mixed Methods and Artificial Intelligence
Source: JMIR Res Protoc. 2023 Oct 16;12:e46652. doi: 10.2196/46652 (PMC10616743; doi:10.2196/46652)
Supplement: Multimedia Appendix 3 [file resprot_v12i1e46652_app3.pdf]

Professional practice	**Please, describe what you do for a living.**<br>**Subquestion 1** : How do you help people who experienced sexual assault and who come to you?<br>**Subquestion 2** : Please, could you describe me a typical day when you are dealing with a victim of sexual violence<br>**Subquestion 3** : What tools, resources, or knowledge (professional or otherwise) do you apply in your practice?
Perception of their patients (notion of victims)	**Please describe the people who come to you after experiencing sexual violence.**<br>**Subquestion 1** : How would you describe the people you see ?<br>**Subquestion 2** : How would you describe the events they have experienced?<br>**Subquestion 3** : What do you think about the place of the notions of "trauma" and "victim" in the reception and support of people who have experienced SA? Do you think those notions are useful ? To whom ?
Patients' needs and adequacy between their needs and the resources accessible to them	**What do you think are the needs of people who have experienced sexual violence and who come to you?**<br>**Subquestion 1** : What would you say about the adequacy between the resources currently in place to help victims of sexual violence and their needs?<br>**Subquestion 2 :** If you had no concerns about resources, how would you improve the system of care for victims of sexual violence?<br>**Subquestion 3** : What do you think of the current care for victims of sexual violence?
Practice perception	**Tell me about how you perceive your practice and the help you provide to victims of sexual violence**<br>**Subquestion 1** : Does your practice always meet the needs of victims? Why or why not ?<br>**Subquestion 2** : How do you feel about helping victims through your practice? Is it adequate? Not enough? Why or why not?<br>**Subquestion 3** : What do you feel produces overwork and limits your practice/the help you can provide to victims (e.g., institutional lack or lack of resources)?
Final questions	Do you have anything you want to add ?<br>What did you think about this interview ?
